# Supplementary material for: Habits, Health and Environment in the Purchase of Bakery Products: Consumption Preferences and Sustainable Inclinations before and during COVID-19
Source: Foods. 2023 Apr 16;12(8):1661. doi: 10.3390/foods12081661 (PMC10138246; doi:10.3390/foods12081661)
Supplement: Supplementary file 1 [file foods-12-01661-s001.zip › foods-2303418-supplementary.pdf]

Supplementary files

Table S1 . Results of the factorial analysis. Total Variance Explained. Source: The authors

Component	Initial Eigenvalues			Extraction Sums of Squared Loadings			Rotation Sums of Squared Loadings		
	Total	% of Variance	Cumulative %	Total	% of Variance	Cumulative %	Total	% of Variance	Cumulative %
1	4.312	26.952	26.952	4.312	26.952	26.952	2.803	17.522	17.522
2	2.265	14.158	41.11	2.265	14.158	41.11	2.422	15.137	32.658
3	1.411	8.82	49.93	1.411	8.82	49.93	2.25	14.059	46.718
4	1.133	7.083	57.013	1.133	7.083	57.013	1.647	10.296	57.013
5	0.894	5.587	62.6						
6	0.857	5.359	67.96						
7	0.788	4.926	72.885						
8	0.682	4.261	77.146						
9	0.635	3.971	81.117						
10	0.609	3.808	84.925						
11	0.553	3.457	88.382						
12	0.472	2.951	91.333						
13	0.431	2.696	94.03						
14	0.39	2.435	96.465						
15	0.321	2.006	98.471						
16	0.245	1.529	100						

Extraction Method: Principal Component Analysis.

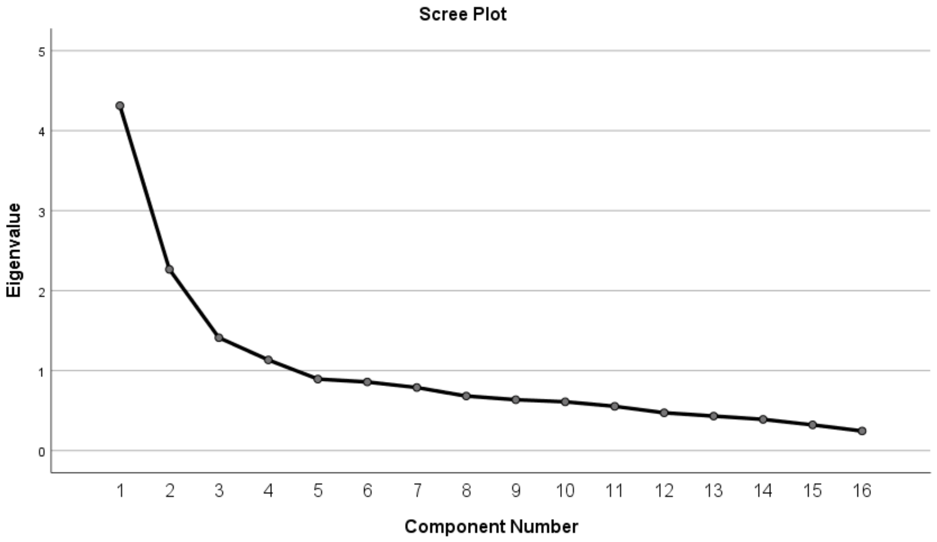

Figure S1 . Scree Plot of factorial analysis. Source: the authors
